# Methanolic *Phoenix dactylifera* L. Extract Ameliorates Cisplatin-Induced Hepatic Injury in Male Rats

**DOI:** 10.3390/nu14051025

**Published:** 2022-02-28

**Authors:** Heba Nageh Gad El-Hak, Hany Salah Mahmoud, Eman A. Ahmed, Heba M. Elnegris, Tahany Saleh Aldayel, Heba M. A. Abdelrazek, Mohamed T. A. Soliman, Menna Allah I. El-Menyawy

**Affiliations:** 1 Zoology Department, Faculty of Sciences, Suez Canal University, Ismailia 41522, Egypt; heba_ahmed@science.suez.edu.eg; 2 Center of Scientific Foundation for Experimental Studies and Research, Ismailia 41511, Egypt; rafeekeltebelnabwy1@gmail.com; 3 Department of Pharmacology, Faculty of Veterinary Medicine, Suez Canal University, Ismailia 41522, Egypt; eman_ahmed@vet.suez.edu.eg; 4 Department of Histology and Cell Biology, Faculty of Medicine, Zagazig University, Zagazig 44519, Egypt; heba.negres@buc.edu.eg; 5 Department of Histology and Cell Biology, Faculty of Medicine, Badr University in Cairo, Cairo 11829, Egypt; 6 Department of Physical Sport Sciences, College of Education, Princess Nourah Bint Abdulrahman University, Riyadh 11671, Saudi Arabia; 7 Department of Physiology, Faculty of Veterinary Medicine, Suez Canal University, Ismailia 41522, Egypt; heba_abdelrazek@vet.suez.edu.eg; 8 Department of Medical Laboratory Sciences, College of Applied Medical Sciences, University of Bisha, Bisha 67614, Saudi Arabia; mohamedtalaat25@yahoo.com; 9 Department of Physiology, Faculty of Medicine, Suez Canal University, Ismailia 41522, Egypt; mennaelmenyawi@med.suez.edu.eg

**Keywords:** antioxidants, cisplatin, date flesh, inflammatory markers, liver, oxidative stress

## Abstract

This study investigated the ameliorative potential of methanolic date flesh extract (MDFE) against cisplatin-induced hepatic injury. Twenty male rats (weighing 180–200 g) were allocated into four groups: control; date flesh (DF) group (oral 600 mg/kg MDFE for 21 days); Cis group (7.5 mg/kg i.p. at day 16); and date flesh/cisplatin (DF/Cis) group (oral 600 mg/kg MDFE for 21 days and 7.5 mg/kg i.p. at day 16). Hepatic biochemical parameters in sera, and inflammatory and oxidant/antioxidant hepatic biomarkers were estimated. Hepatic histological changes and the immunohistochemistry of cyclooxygenase-2 (COX-2), nuclear factor kappa B (NF-κB), and alpha smooth muscle actin (α-SMA) were assessed. Pretreatment with MDFE decreased Cis-triggered liver biochemical parameters, oxidative stress, inflammatory biomarkers, and histological damage. Moreover, MDFE treatment reduced Cis-induced hepatic NF-κB, COX-2, and α-SMA protein expression. MDFE exerted a hepatoprotective effect when used concomitantly with Cis. Its effect was mediated via its antioxidant and anti-inflammatory ingredients.

## 1. Introduction

Chemotherapeutic agents have widely been used to slow and/or stop the development of cancer cells [1]. Cisplatin (Cis), cis-diammineplatinum (II) dichloride, is a platinum-based drug which is one of the most effective anticancer agents. It has been widely used to treat testicular, ovarian, cervical, bladder, neck, and lung cancers [2]. Cisplatin’s cytotoxic influences on cancer cells are thought to be attributable to its interaction with DNA, resulting in the formation of covalent adducts between certain DNA bases and the platinum compound [3]. Therefore, Cis produces acceptable results in treating later cancers [4].

Cisplatin is transported into cells via an ATP-binding cassette transporter MDR1, which is a P-glycoprotein in nature [5]. The transport of Cis to mammalian cells is partly governed by copper transporter Ctr1 [6], which increases its absorption, and membrane proteins such as TMEM205 may limit its entry into cells [5]. Once Cis enters the cell, water molecules displace its chloride atoms, making it a potent electrophile that can react with nucleic acids’ nitrogen atoms and proteins’ sulfhydryl groups. Cisplatin could block cellular division and trigger apoptosis by binding purine N7′s reactive center, resulting in DNA damage and apoptotic cell death [2]. Oxidative stress induced by Cis is another mechanism for its toxicity. Cisplatin targets mitochondrial protein, causing its loss; inhibits calcium uptake; and reduces the potential of the mitochondrial membrane, which triggers lipid peroxidation and apoptosis [7]. Moreover, Cis activates the Jun amino-terminal kinase (JNK) pathway and p38 mitogen-activated protein kinase (MAPK) via interacting with p53, which stimulates the transcription of proapoptotic genes NOXA and PUMA [8].

Cisplatin, similarly, to many other chemotherapeutic agents, does not distinguish between cancerous and non-cancerous cells. Therefore, myriad side effects have been recorded for chemotherapy [9]. Cis has been reported to induce nephrotoxicity, hepatotoxicity, and cardiotoxicity [10]. Cisplatin-induced hepatotoxicity has been recorded in several case reports, triggered by the generation of reactive oxygen species, weakening of the antioxidant defense system, and cascades of inflammatory reactions [11,12]. Hepatotoxicity is a serious problem that occurs concomitantly with treating cancer patients with Cis [13]. Cisplatin enters the cell via passive transport. It undergoes hepatic metabolism and is subjected to biotransformation by the cytochrome P450 (CYP450) enzyme complex into cytochrome P450 2E1 (CYP2E1), which is responsible for cisplatin hepatic injury [14]

The adverse effects of chemotherapeutic agents, including Cis, represent an urgent need to provide chemotherapy regimens with supportive drugs and/or supplements to counteract their adverse effects [15]. Several natural medicinal plants and drugs can be used and potentially ameliorate Cis-induced side effects.

Medicinal plants have historically been the preferred options for ameliorating pathological side-effects, as their own side effects are normally minor [16]. *Phoenix dactylifera* (date palm) is one of these plants that has been documented to treat liver-disease-related clinical symptoms in Arabian, Middle Eastern, and Northern African countries [17]. Middle Easterners have traditionally believed that eating dates on an empty stomach, especially in the morning, protects the liver from the action of toxic materials [18]. Dates contain biologically important phenolic acids, such as vallinic acid and syringic acid, which are not usually found in cranberries or other dried fruits, such as pears, prunes, and apricots. The phenolic content and related polyphenol content in dates are correlated with cultivation soil, development and growth stages, environmental exposures, and pests [19]. Okwuosa et al. [20] showed that MDFE protected the liver from a thioacetamide chemical insult. Another study by Al-Ghasham et al. [21] confirmed the hepatoprotective properties of date fruit extract against aflatoxin B1-induced hepatotoxicity. Date palm flesh extracts are rich in compounds that possess anti-inflammatory, antioxidant, and anti-carcinogenic properties in both in vivo and in vitro systems [22].

Date flesh extract was proven to produce various molecular and cellular modulations that were involved in its hepatoprotective effect. It was associated with reducing the expression of nuclear factor kappa B (NF-κB), which is a key transcription factor in the activation of hepatic stellate cells. It also caused the inhibition of translocation from the nucleus, eventually preventing the expression of inflammatory cytokines such tumor necrosis factor-alpha (TNF-α), IL-1β, and interleukin-6 (IL-6); and inducible nitric oxide synthase and cyclooxygenase-2 (COX-2), which are involved in the process of fibrogenesis [23]. Transforming growth factor beta (TGFβ) is downregulated via date flesh extract [24], which goes on to downregulate extra cellular matrix (ECM) by inhibiting matrix metalloproteinases (MMPs) and simultaneously upregulates TIMPs [25], leading to failure of ECM formation and accumulation of collagen in hepatic tissue [26].

The widespread consumption and availability of date palms as botanical and medicinal plants suggest the importance of this plant in human healthcare. The liver is where most drug and xenobiotic metabolism takes place, so it influences their toxicity and effects. The present work investigated the possible protective effects of MDFE on Cis-induced liver injury. For this purpose, hepatic oxidative stressors (malondialdehyde (MDA) and protein carbonyl content (PCC)), antioxidants (superoxide dismutase (SOD) and reduced glutathione (GSH)), and metabolic cofactors (nicotinamide adenine dinucleotide phosphate (NADPH) and alcohol dehydrogenase (ADH)) were assessed. Hepatic inflammatory biomarkers (interleukin-12 (IL-12), interleukin-10 (IL-10), and Cox-2) and hepatic serum biomarkers (liver enzymes, total protein, albumin, and globulin) were also measured. The antioxidant and hepatoprotective effects of MDFE were evaluated via manipulating NF-κB, interleukins, COX-2, and oxidative stress-triggering pathways, with special emphasis on their influences on alpha smooth muscle actin (α-SMA) as a hepatic fibrosis indicator.

## 2. Materials and Methods

### 2.1. Drug and Plant Extraction

Cisplatin (CAS Number:15663-27-1) was purchased from Sigma Chemical Company (St. Louis, MO, USA). *Phoenix dactylifera* (date palm) (100% organic) was purchased from a local market in Saudi Arabia. Powdered aerial parts of dates were extracted with methanol. Briefly, 100 g of date flesh was ground into a fine powder. The powder was treated with n-hexane for defatting and then extracted three times with 500 mL methanol at room temperature for 24 h with magnetic stirring. The methanolic crude extracts were obtained by filtration, then centrifuged at 6000 G for 30 min at 3 °C. The obtained supernatant was concentrated under low pressure at 40 °C for 1 to 2 h using a rotary evaporator [27]. The extract, in the form of solid dry extract, was kept at −4 °C in carboxy methyl cellulose (CMC) 1:5 (*w*/*v*) to be ready for use.

### 2.2. Characterization of Compounds in the Methanolic Date Palm Extract Using LC-MS/MS Analysis

Date flesh extract (50 mg) was dissolved in reconstitution solvent (water: methanol: acetonitrile, at a 2:1:1 ratio), vortexed, and sonicated to obtain 1 µg/µL. The diluted samples were subjected to 10,000 rpm centrifugation for 5 min. Subsequently, 10 µL of each supernatant was injected [28].

An ExionLC system (AB Sciex, Framingham, MA, USA) connected to a Triple TOFTM 5600+ Q-TOF-MS system was used to perform secondary metabolite analysis (AB SCIEX, Concord, Canada). An Xbridge C18 column (3.5 µm, 2.1 × 50 mm) (Waters Corporation, Milford, MA, USA) was used for analysis. The gradient solutions consisted of acidified 5 mM ammonium formate in 1% methanol at pH = 3.0 and pH = 8.0 for the positive and negative modes, respectively. The organic solution was 100% acetonitrile. The gradient consisted of 10% organic for 20 min; then 90% organic for 5 min; 10% organic for 3 min; and then 90% organic for column equilibration [29]. The secondary molecules were recognized depending on *m*/*z* and the MS/MS pattern by aligning their fragmentation patterns with those available in reference databases. Moreover, molecular formulae, adducts, and retention times were validated.

### 2.3. Animals

Twenty adult male Wister rats (weighing 180–200 g) were obtained from the Zoology Department Animal House (Ismailia, Egypt). Animals were accommodated to laboratory conditions (24 ± 2 °C and 55–60% humidity) in normal light and dark cycles. Rats were fed standard rat pellets and provided tap water ad libitum. The experiment began after a two week adaptation period. Following the National Research Council Guide for the Care and Use of Laboratory Animals [30], the experimental animals were handled, kept, and used according to the EU Directive 2010/63/EU, in compliance with ARRIVE guidelines. All experimental procedures received the approval of the Ethics Committee at the Veterinary Medicine, Suez Canal University, Ismailia, Egypt (20211043).

### 2.4. Experimental Design

Twenty adult male Wister rats weighing 182–200 g were allocated equally into four groups (5 rats per group). The control group was given CMC daily for 21 days as a vehicle and was injected with saline i.p. on the 16th day of the experiment. The date flesh (DF) group received 600 mg/kg MDFE in CMC daily for 21 days and was injected with saline i.p. on the 16th day of the experiment. The Cis group was administered CMC as a vehicle daily for 21 days and injected with saline Cis (7.5 mg/kg) i.p. on the 16th day of the experiment [31]. The date flesh/cisplatin (DF/Cis) group was treated with 600 mg/kg MDFE in CMC daily for 21 days and with saline Cis (7.5 mg/kg) i.p. on the 16th day of the experiment.

### 2.5. Sampling

Rats were made to fast overnight at the end of the experiment, weighed, and anaesthetized via an i.p. urethane injection (1.2 g/kg;) for anesthesia [32]. Blood samples were obtained from the retro-orbital plexus; serum was separated and kept at –80 °C. The animals were euthanized; then, the liver was excised, weighed, and washed with ice-cold phosphate-buffered saline. Each rat liver was divided into two parts. The first part was kept at –80 °C for further homogenization to assess oxidative stress markers, antioxidant activity, and ELISA. The second part was fixed in 10% neutral buffered formalin for histopathological and immunohistochemical investigations. The liver tissues stored at −80 °C were homogenized (10% *w/v*) in ice-cold sodium phosphate buffer (0.01 M, pH 7.4) containing 1.15% KCl. The homogenates were subjected to centrifugation for 20 min at 3000 rpm and 4 °C; the supernatant was harvested and kept at −80 °C.

### 2.6. Relative Liver Weights

The relative liver weights were calculated according to Mossa et al. [33]: relative liver weight = [liver weight/body weight] × 100.

### 2.7. Serum Biochemical Analysis

The activities of liver injury biochemical markers, including aspartate aminotransferase (AST) and serum alanine aminotransferase (ALT), were estimated using the Infinity TM Reagent kit (Thermo, TR70021, Auburn, AL, USA). The kinetic assay of AST was carried out according to the IFCC method [34]. The primary wavelength (WL) applied in AST and ALT kinetic assays was 340 nm and the secondary WL was 405 nm, with linearity and sensitivity equivalent to 450 U/L and 0.573 ΔmA/min per U/L, respectively. The system lag was 30 min. The ratio of AST to ALT, as a guideline, was used to diagnose liver disease according to Liu et al. [35]. Levels of gamma-glutamyl transferase (GGT), a bile duct lesion indicator, were determined using the kinetic photometric method according to Szasz [36], where γ-glutamyl-p-nitroanilide in ammediol-HCl buffer (pH 8.2) was employed as a colorless substrate at 25 °C to obtain optimum reaction conditions. The GGT transferred the gamma-glutymyl group of the substrate to an acceptor to form p-nitroaniline (a colored product). A computerized system monitored over 60 readings/minute at absorbance 410 nm. The rate of GGT activity was directly proportional to the absorbance change rate. The quantitative determination of albumin levels in sera was performed according to de Leeuw-Israel et al. [37]. The 2-(4′-hydroxybenzeneazo) benzoic acid dye was applied in this method, and albumin was detected by the spectro-calorimetric method at WL 510 nm. Total protein was determined using a calorimetric kit (#MBS2540455, My BioSource, Inc., San Diego, CA, USA). The assay depends on the reduction of Cu^++^ to Cu^+^, in an alkaline environment, by the sample protein. The reduced Cu+ reacts with bicinchoninic acid, producing a purple product, the optical density (OD) of which was read at 562 nm. The inter-assay CV and the intra-assay CV were 4.72% and 2.06%, respectively. Globulin was determined by subtracting albumin from total protein after the calorimetric assays according to Debro et al. [38].

### 2.8. Hepatic ADH and NADPH

Alcohol dehydrogenase activity was determined photometrically, according to Skurský et al.’s method [39]. That protocol is based on ADH generating power via NADH from NAD and n-butanol. The generated NADH is capable of enzymatically reducing the yellow substrate p-nitrosodimethylaniline into a colorless compound. Fading of the yellow color in the recycling reaction is indicative of ADH activity, which is measured by the kinetic method. NADPH activity was assessed according to Wagner and Scott’s method [40]. The assay is based on monitoring a glucose dehydrogenase cycling reaction in which NADPH reduces a formazan reagent (MTT) into a colored product. We calorimetrically measured said compound at WL 570 nm in heated tissue extract that was prepared in the dark.

### 2.9. Oxidative Stress Markers, Antioxidant Activity, Interleukin-12, and Interleukin-10

Liver lipid peroxidation, indexed as the MDA concentration, was estimated using a colorimetric MDA Assay Kit (catalogue number ab233471, Abcam, Waltham, MA, USA). The reaction was based on the interaction of thiobarbituric acid (TBA) with MDA to form a reactive species of TBA, which was estimated at 695 nm absorbance [41]. Additionally, liver protein oxidation was determined by measuring the PCC using a calorimetric assay kit (catalogue number ab126287, Abcam, UK). The test is based on the reaction of DNPH with protein carbonyl DNP hydrazones, which were quantified at 375 nm absorbance [42]. Hepatic copper–zinc SOD activity was calorimetrically determined using an assay kit (catalogue number ab65354, Abcam, UK) at 560 nm [43]. Liver GSH activity was quantified using a kinetic assay kit (catalogue number MAK364-1KT, Merck, Darmstadt, Germany) and absorbance at 450 nm [43]. The hepatic levels of IL-10 and IL-12 were assessed using specific ELISA Kits (CSB-E07364r, CUSIBIO, Houston, TX, USA; and UniProtKB-P29456, UniProt, Washington, DC, USA, respectively), following to the manufacturers’ instructions.

### 2.10. Hepatic Histology and Immunohistochemistry

Neutral buffer formalin-fixed livers were embedded in paraffin wax for 24 h, cut into 5 µm sections, and stained with hematoxylin–eosin (H&E). The livers were histological examined, blindly, for pyknotic cells, hydropic degeneration, focal necrosis, and inflammation. The degree of liver damage was assessed using a score from 1 to 3 as follows: (1) mild; (2) moderate; (3) severe, in accordance with Brunt et al. [44].

Immunohistochemistry (IHC) for NF-κB, α-SMA, and cyclooxygenase 2 (COX-2) was performed. As with other sets of paraffin-embedded tissues, they were cut into 5 µm thick sections, deparaffinized in xylene, and rehydrated with aqueous alcohol solutions [45]. For antigen retrieval, tissue slices were immersed in 0.01 M sodium citrate buffer (pH 6.0) and subjected to boiling for 5 min twice to hasten immunoreactivity. Slides were cooled, and phosphate-buffered saline (pH 7.2) was added. The samples were incubated at 4 °C overnight with their various primary antibodies (Santa Cruz Biotechnology Inc., Santa Cruz, CA, USA) at 1:300 concentrations. After washing the slides, incubation with secondary polyvalent antibody was performed. Finally, the tissue slices were washed, and the immunoreactivity was visualized using Genemed Power-Stain, 1.0 Poly HRP DAB kit (Sakura Finetek Inc, Torrance, CA, USA). Tissue slices were washed, and counterstaining with Mayer’s hematoxylin was performed. The percentages of IHC staining were scored as follows: strong when positive staining was in 50–75% of the examined areas; moderate when positive staining was in 25–50% of the examined areas; mild when positive staining was in <25–50% of the examined areas; and very strong when positive staining was in >75% of the examined areas. The areas of IHC positivity in the liver for eight random images of each slide were assessed in ImageJ software. A histologist blinded to the tested animal groups assessed the grading of liver IHC using a light microscope.

### 2.11. Statistical Analysis

Data were statistically analyzed using SPSS software. One-way analysis of variance (ANOVA) was used to assess differences between tested groups, followed by Duncan’s multiple comparison tests. Statistical analyses of histological and IHC staining intensity scores were performed with non-parametric Kruskal–Wallis tests. Results are presented as the means ± standard errors of means (SE); *p*-values < 0.05 were considered statistically significant.

## 3. Results

### 3.1. LC-MS/MS Analysis

Metabolites found in both positive and negative modes showed that flavonoids, flavanones, anthocyanidin-3-O-glycosides, flavonoid-7-O-glycosides, cinnamic acids, hydroxycinnamic acids, and flavonoid-3-O-glycosides were present in the methanolic extract of date flesh (Table 1). All those compounds possess antioxidation and free radical scavenging abilities.

### 3.2. Relative Liver Weights

**Figure 1:**
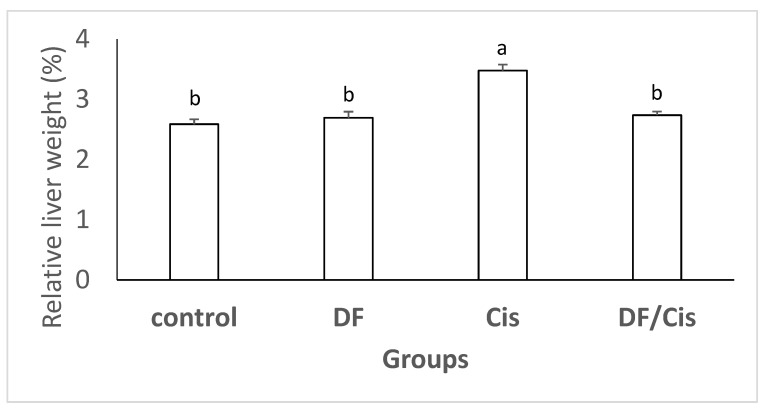
Relative weights of liver of male rats treated with methanolic extract of date flesh (MDFE) and/or cisplatin (Cis). Values are presented as the means ± SE. (*n* = 5). Means with different letters (a,b) are significantly different, *p ≤* 0.05.

The relative liver weights of rats treated with Cis were greater than those of control rats *(p* ≤ 0.05). The DF/Cis group’s liver weights were decreased *(p* ≤ 0.05) compared with the Cis group. The DF group was non-significantly different from the control group (Figure 1).

### 3.3. Serum Biochemical Analysis

Table 2 reveals that the values of serum ALT, AST, and GGT increased (*p* ≤ 0.05) in rats injected with Cis compared with the control. Meanwhile, serum albumin declined (*p* ≤ 0.05) in rats injected with Cis in comparison with the control rats. Pretreatment with MDFE before injection with Cis significantly *(p* ≤ 0.05) reduced the expression levels of ALT, AST, and GGT, but increased the albumin level in the Cis group. The globulin level exhibited non-significant alterations among the test groups.

### 3.4. Hepatic ADH and NADPH

Both liver ADH and NADPH exhibited a significant reduction *(p* ≤ 0.05) in the Cis administered group compared with the control rats. The pretreatment with MDFE significantly *(p* ≤ 0.05) elevated ADH and NADPH as compared with the Cis group (Table 2).

### 3.5. Oxidative Stress Markers, Antioxidant Activity, Interleukin-12, and Interleukin-10

Lipid peroxidation end products (MDA) and PCC were increased *(p* ≤ 0.05) in the Cis-administered group when compared with the control group. However, the Cis group decreased *(p* ≤ 0.05) liver SOD and GSH expression compared with the control group. Pretreatment with MDFE significantly decreased (*p* ≤ 0.05) liver MDA and PCC levels, whereas it increased SOD and GSH levels (Table 3). MDFE administration induced significant *(p* ≤ 0.05) promotion in SOD activity compared with the control group.

Administration of Cis significantly increased (*p* ≤ 0.05) the hepatic contents of IL-10 and IL 12. Meanwhile, pretreatment with MDFE before Cis injection significantly decreased (*p* ≤ 0.05) the hepatic IL-10 and IL-12 concentrations when compared with the Cis group. MDFE administration induced significant *(p* ≤ 0.05) promotion of hepatic IL-10 levels when compared with the control.

### 3.6. Histology and Immunohistochemistry of Liver

**Figure 2:**
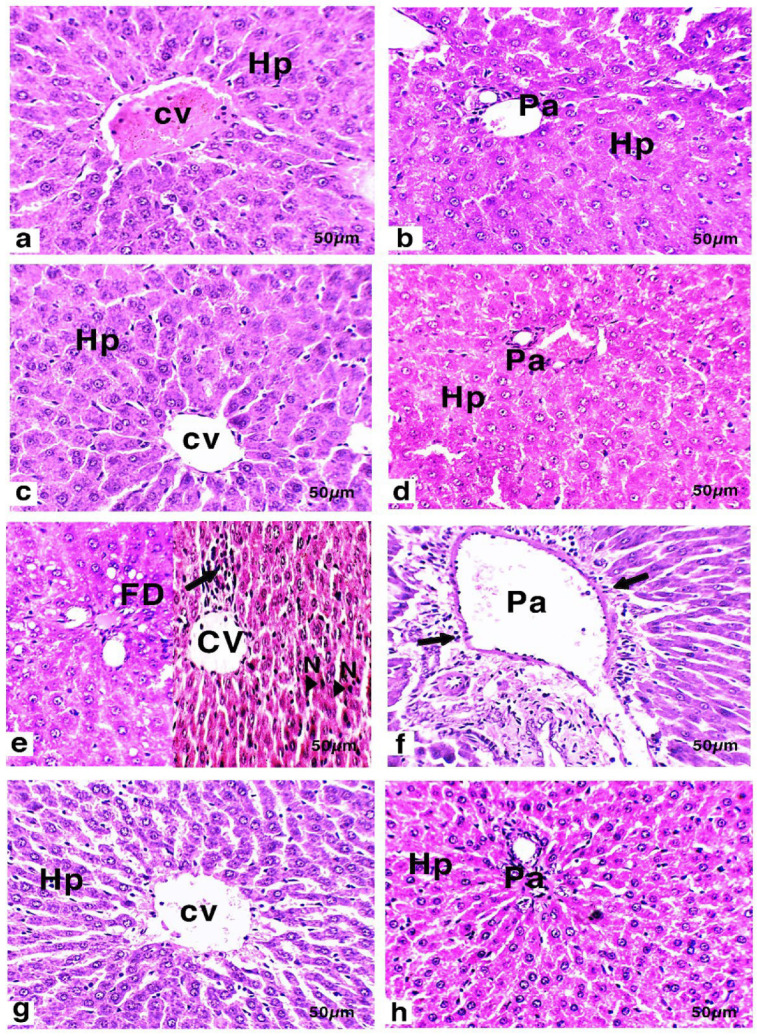
(**a**,**b**) Liver section of a control rat. (**c**,**d**) Liver sections of rats treated with methanolic extract of date flesh (DF) showed a normal histological structure of hepatocytes (Hp) surrounding the central vein (CV) and the portal area (Pa). (**e**,**f**) Liver sections of rats treated with cisplatin (Cis) showed mild fatty degeneration (FD), focal necrosis (N) near the central liver region, and infiltration of the inflammatory area (arrow) surrounding the portal area (Pa) and (CV). (**g**,**h**) Liver sections of the group pretreated with methanolic extract of date flesh before injection with cisplatin (DF/Cis) exhibited normal histological structure of hepatocytes (Hp) surrounding the central vein (CV) and the portal area (Pa). Scale bar = 50 µm (H&E, 200X).

Histological images of the livers of rats from the various groups are presented in Figure 2 and Table 4. Light microscopic observations of liver tissue from the control (Figure 2a,b) and DF (Figure 2c,d) groups demonstrated normal histological structure. The hepatocytes appeared to be normal large polygonal cells with eosinophilic cytoplasm and prominent round nuclei, and the few hepatic sinusoids were spaced out in-between the hepatic cords where Kupffer cells were finely arranged. The liver sections of Cis-treated rats exhibited histological damage (Figure 2e,f). The most pronounced histological abnormality observed was the mild fatty degeneration of hepatic cells. The liver tissues revealed the existence of severe and dense infiltration of inflammatory cells, specifically in the parenchymal, intermediate and periportal areas, in addition to the portal spaces. In contrast, groups receiving MDFE and Cis exhibited less damage than that induced in the Cis group (Figure 2g,h). Hepatocytes which appeared normal surrounded the central vein; additionally, mild infiltration of the inflammatory cells was noticed.

Table 4 shows the mean histological scores of the groups. Hepatic histological changes in the control and DF groups were not detected in any rat. The intensities of necrosis and hydropic and fatty degeneration were characterized as mild (degree 1) and moderate (degree 2) in the Cis group. Inflammatory infiltration with lymphocytes in the hepatic parenchyma was regarded as moderate or severe (degrees 2 and 3), occurring principally in the Cis group. On the other hand, the degrees of necrosis, hydropic degeneration, and lymphocyte inflammatory cell infiltration in the liver parenchyma were mild (degree 1) in the DF/Cis group.

**Figure 3:**
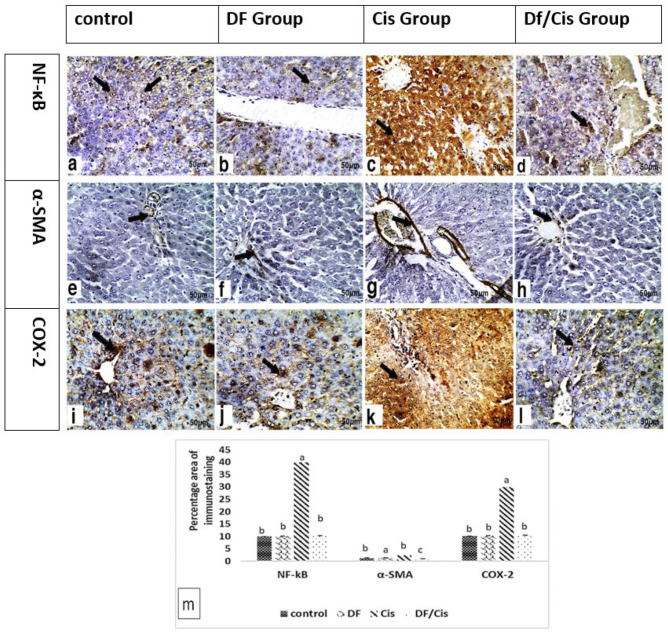
(**a**–**l**) Immunoexpression of nuclear factor kappa B (NF-κB) in the cytoplasm, alpha smooth muscle actin (α-SMA) in the portal area, and cyclooxygenase 2 (COX-2) in the cytoplasm of the stained sections of the liver of rats belonging to the control group and methanolic extract of date flesh (DF)-treated group. (**a**,**b**,**i**,**j**) Liver sections of control rats and rats treated with methanolic extract of date flesh (DF) showed mild brown immunoexpression of nuclear factor kappa B (NF-kB) and cyclooxygenase 2 (COX-2) in the cytoplasm of hepatocytes (arrow). (**e**,**f**) Liver sections of control rats and the DF group showed mild brown immunoexpression of α-SMA in the portal area (arrow). (**c**,**k**) Liver sections of rats treated with cisplatin (cis) showed severe brown immunoexpression of nuclear factor kappa B (NF-κB) and cyclooxygenase 2 (COX-2) in the cytoplasm of hepatocytes (arrow). (**g**) Liver sections of rats treated with Cis showed severe brown immunoexpression of α-SMA in the portal area (arrow). (**d**,**l**) Liver sections of the group pretreated with methanolic extract of date flesh before injection with cisplatin (Cis/DF) showed mild brown immunoexpression of nuclear factor kappa B (NF-κB) and cyclooxygenase 2 (COX-2) in the cytoplasm of hepatocytes (arrow). (**h**) Liver sections of Cis/DF-group rats showed mild brown immunoexpression of α-SMA in the portal area (arrow) (200×, scale bar = 50 µm). (**m**) Histogram of the mean percentage areas of NF-κB, α-SMA, and COX-2 protein expression in the hepatocytes of different groups (*n* = 5). Different superscript letters denote significant differences at *p* ≤ 0.05.

Liver immunohistochemical assessments of the inflammation pathway in control and treated rats are presented in Figure 3 in the form of brown cytoplasmic coloration of the hepatocytes and the portal area. Immunohistochemical detection of NF-κB and COX-2 in liver sections revealed mild expression in control and DF group hepatocytes, respectively, in addition to the immunohistochemical detection of α-SMA in liver sections, which revealed mild expression in control and DF group hepatic portal areas. Remarkably, treatment with Cis triggered significantly higher expression (*p* ≤ 0.05) of NF-κB and COX-2, as indicated by strong positive brown cytoplasmic staining in the hepatocytes, in addition to the strong immunohistochemical detection of α-SMA in the portal area. The pretreatment Cis group with MDFE revealed mild expression of COX-2 and NF-κB in the cytoplasm of hepatocytes and mild expression of α-SMA in the hepatic portal area compared with the Cis group.

## 4. Discussion

Cisplatin is a potent anticancer drug used as a chemotherapeutic agent. However, it is often limited by its undesirable side effects, such as hepatic injury, which occurs with a high dose, even after a short time [2]. The protective role of MDFE extract in Cis-induced hepatic injury has been examined in this study.

The LC–MS analysis of MDFE showed a wide range of phenolic and flavonoid compounds, which contribute to several beneficial health functions [46]. Some of the main potential health benefits of phenolics and flavonoids are their antioxidant activities [47]. Among the ingredients, flavonoid-7-O-glycosides, anthocyanidin-3-O-glycosides, traumatic acid, *p*-coumaric acid, ferulic acid, and kaempferol-3-O-alpha-L-rhamnoside are flavonoids and phenolic compounds that possess strong 2,2-diphenyl-1-picrylhydrazyl (*DPPH)* scavenging activity [48]. Phlorizin is a phenylpropanoid ingredient of MDFE that possesses antioxidant potential [49]. The results presented here are in the line with those of Djaoudene et al. [50], who demonstrated that total phenolic and flavonoid contents of MDFE are closely related to its ability to eliminate free radicals. The scientific community has only recently investigated the antioxidant properties of date fruits. Apart from their nutritional value, dates are rich in phenolic compounds with antioxidant activity [51].

The unique concentrations of ingredients in MDFE make it superior to other fruits, as stated by Guo et al. [52], who found that dates had the second strongest antioxidant activity among 28 fruits by using the fluorescence recovery after photobleaching (FRAP) test. Ferrous iron (Fe^2+^) can generate free radicals from peroxides by the Fenton reaction and is implicated in many diseases. Reducing the Fe^2+^ available for the Fenton reaction would protect against oxidative damage. Nevertheless, the mechanism of date extract involves suppressing hydroxyl radicals, according to hydroxyl radical assay, wherein the extract provided the same complete inhibition as TBA (4.0 mg/mL) [53]. Considering data obtained with different antioxidant methods, it appears that the ability of MDFE to avert the free radicals generation through iron chelation was the major antioxidant mechanism accountable for the observed antioxidant activity.

Increased hepatic weight is obviously associated with liver injury. The administration of Cis to experimental rats induced significant elevation in relative liver weights. Our results are in agreement with those of Sangha et al. [54]. The ability of MDFE to ameliorate the increase in hepatic weight demonstrated its ability to reduce Cis-induced hepatic injury.

Findings of the biochemical assay demonstrated elevations in ALT, AST, AST/ALT, and GGT levels in rats injected with Cis compared with the control rats. These findings confirmed the hepatic injury induced by Cis administration, whereas elevation in the serum levels of these enzymes is an obvious sign of hepatocellular injury [55]. These results were supported by the increased hepatic expression of MDA and PCC. The increased oxidative load by Cis enhanced the depletion of liver enzymatic (SOD) and non-enzymatic (GSH) antioxidants. Therefore, free radicals were increased and reacted with macro-cellular components: lipids and proteins [55]. Increased MDA has been associated with extensive hepatocyte membrane lipid peroxidation, leading to hepatocyte enzyme contents leaking into circulation [56]. These results are in agreement with Palipoch and Punsawad [57]. Several studies have identified the hepatotoxic effect of Cis [58,59,60].

Liver NADPH contents were significantly reduced in the Cis-administered group. Hepatic NADPH has a reducing equivalent for oxidation–reduction reactions tangled in free radical scavenging, in addition to allowing the regeneration of GSH [61]. A reduction in hepatic ADH, an oxidoreductase enzyme, may occur with a reduction in NADPH, which is product of alcohol reduction via an enzymatic reaction [62].

The depletion of antioxidants and the increased oxidative stress in the Cis group caused functional impairments to hepatocytes in protein synthesis (albumin specifically) [57], which was reduced in the current study. Hepatic histological changes in the Cis group were attributed to the oxidative stress and lipid peroxidation which were noted in the present study. Failure of the antioxidant defense to protect against hepatocyte membrane peroxidation was manifested by reduced hepatic SOD and GSH levels. Oxidative stress has been closely associated with inflammatory pathways. A sustained and unregulated imbalance between the creation of free radicals and/or their removal by antioxidants prompts the generation of proinflammatory cytokines. The latter provokes further free radical production, resulting in a vicious cycle. NF-κB, a member of the inducible transcription factor family, is promoted via oxidative stress [63]. NF-κB is the main transcription factor of M1 macrophages and is compulsory for inducing a large number of inflammatory genes: IL-1β, IL-6, IL-12, TNF-α, and COX-2 [49]. On the other hand, M2 macrophages synthesize anti-inflammatory cytokines, such as IL-10 [64]. Therefore, NF-κB inhibition is a goal for the treatment of numerous inflammatory diseases. This study has showed that Cis administration promotes hepatic NF-κB protein expression, which promotes IL-12 production and COX-2 expression while downregulating IL-10. It is clear that Cis promotes M1–M2 macrophage polarization, which involves the hepatic inflammation signaling pathways [65]. Similarly, Shaker et al. [66] concluded that Cis leads to elevated IL-12, which supports its hepatic inflammatory reaction.

The alpha smooth muscle actin expressed in hepatic stellate cells and vascular smooth muscle cells is a cornerstone of liver fibrosis. It is influenced by COX-2 production, which is crucial in the pathogenesis of hepatic inflammation [67]. The upregulation of COX-2 has been detected in various hepatic inflammatory diseases, including liver fibrosis [68,69,70]. The present study demonstrated an increase in α-SMA immunoprotein expression in the Cis group, which seemed to be influenced via NF-κB overactivity, oxidative stress, and COX-2. This increment is a predictor for future Cis-induced hepatic fibrosis.

Pretreatment with methanolic flesh date extract modulates the oxidative damage caused via the promotion of antioxidant activity of SOD and GSH contents, which reduces lipid peroxidation and PCC after Cis administration. Similarly, Khalid et al. [71] showed that MDFE increased the antioxidant defense mechanism in rats, and provided evidence of its possible protective role in diseases that produce free radicals. Additionally, El-Far et al. [72] showed that MDFE triggered a reduction in MDA levels, with a powerful free radical eliminating ability. The amelioration of oxidative stress properties may be attributed to the ingredients detected in MDFE by LC-MS. Flavonoids [73], flavanones [74], anthocyanidin [75], flavonoid-7-O-glycosides, flavonoid-3-O-glycosides [76], cinnamic acids [77], and hydroxycinnamic acids [78] have previously been demonstrated to exert antioxidant and hepatoprotective effects. The latter could possibly reduce membrane lipid peroxidation, thereby maintaining hepatocytes’ membrane integrity and preventing the leakage of enzymes into the blood. This was manifested by reduced serum levels of ALT, AST, and GGT. The amelioration of hepatic injury via MDFE led to improvements in the liver synthesis of plasma proteins (total protein, albumin, and globulin). Okwuosa et al. [20] and Hussein et al. [79] reported the same effect of MDFE against different experimentally induced liver injuries in rat models.

The antioxidant effect of MDFE prevents hepatic NADPH depletion in oxidation–reduction reactions, and the regeneration of GSH. Additionally, the restoration of hepatic ADH may induce observed improvements in NADPH levels as a product of ADH-mediated alcohol reduction [62]. Additionally, active MDFE ingredients cause M1–M2 macrophage polarization, successfully resulting in the promotion of IL-10, which mitigates Cis-induced hepatic inflammatory changes, that seems to be NF-κB and COX-2-dependent [80]. The previous changes abrogated the expression of α-SMA, which later reduced the incidence of hepatic fibrotic inflammation.

Histological assessment of the liver revealed Cis-induced hepatic damage. The observed lesions were destructive to most hepatocytes, in addition to leukocyte infiltrations in the portal areas and around central vein. According to Hakiminia et al. [81], the existence of inflammatory cells in liver tissue is attributable to the interactions of Cis with the enzymes and proteins of the interstitial liver tissue—meddling with the antioxidant defense machinery and causing the generation of reactive oxygen species, which then may induce an inflammatory response. Hydropic degeneration is a response to excess water accumulation inside cells, which leads to the accretion of sodium, and consequently, the entry of water, as explained by Rubin et al. [82]. Some hepatocytes of the Cis-treated rat livers exhibited focal necrosis, which may have been due to Cis-induced DNA synthesis inhibition, which is essential for the maturation and growth of the liver.

The present study declared the efficacy of MDFE at 600 mg/kg in counteracting Cis-induced hepatic injury in rats. There is no clinical evidence of effects of MDFE in humans, especially regarding liver disease. It may be beneficial to use said extract in humans as a hepatoprotection during Cis chemotherapy. This would be safe and cheap. The human dose for MDFE would be equivalent to 97.2 mg/kg body weight according to Paget and Barnes [83].

## 5. Conclusions

Cisplatin-induced hepatic injury and inflammation manifested as elevations in enzymatic indices and histological lesions. These effects were mediated via the induction of oxidative stress, which promoted the NF-κB inflammatory cascade, including IL-12 and Cox-2, while reducing IL-10. Pretreatment with MFDE markedly ameliorated Cis-induced hepatic injury due to its active antioxidant ingredients. MFDE reduced NF-κB activity, downregulating IL-12 and COX-2. It also prevented the overproduction of reactive oxygen species, reduced inflammation, and reduced further hepatic fibrotic changes via α-SMA. Therefore, MDFE is recommended for use in humans to mitigate Cis-induced injury, especially since it is cheap and widely available.

## Figures and Tables

**Table 1:** List of identified metabolites in both positive and negative modes.

#	RT (min)	Name	Precursor *m*/*z*	Error ppm	Adduct	Formula	Class	MS/MS Spectrum
1	1.22	3′,4′,5,7-tetrahydroxyflavanone	289.0819	−2.2	[M+H]^+^	C_15_H_12_O_6_	Flavanones	127.04 [C_6_H_6_O_3_]+H^+^, 135.04 [C_8_H_8_O_2_-H]^+^, 149.06 [C_9_H_7_O_2_+H]+H^+^
2	1.22	4′,5,7-Trihydroxy-3-methoxyflavanone	303.095	0.8	[M+H]^+^	C1_6_H_14_O_6_	Flavonoids	122.1 [C_7_H_6_O_2_]^+^, 136.1 [C_7_H_4_O_3_]^+^, 166.1 [C_9_H_10_O_3_]^+^, 213.1 [C_13_H_9_O_3_]^+^, 256.1 [C_15_H_10_O_4_+H]+H^+^
3	1.27	Kojibiose	341.1921	22.7	[M-H]^−^	C_12_H_22_O_11_	Fatty Acyls	89.1 [C_3_H_4_O_3_]+H^+^, 161.1 [C_6_H_10_O_5_-H]^+^, 179.1 [C_6_H_11_O_6_]^+^, 221.1 [C_8_H_13_O_7_]^+^
4	1.27	trans-Cinnamate	147.0445	−97.6	[M-H]^−^	C_9_H_8_O_2_	Cinnamic acids	87.1 [C_7_H_6_-_2_H]-H^−^, 103.028 [C_8_H_7_]^−^, 129.1 [C_9_H_7_O-H]-H^−^
5	1.39	Myricetin	319.037	1.4	[M+H]^+^	C_15_H_10_O_8_	Flavonols	137.1 [C_7_H_4_O_3_]+H^+^, 200.1 [C_11_H_5_O_4_-H]^+^, 214.1 [C_12_H_6_O_4_]^+^, 229.1 [C_12_H_6_O_5_-H]^+^, 301.1 [C_15_H_9_O_7_]^+^
6	1.53	Xanthine	151.0233	12.4	[M-H]^−^	C_5_H_4_N_4_O_2_	Xanthines	71.1 [C_2_HNO_2_]^+^, 108.1 [C_4_H_3_N_3_O-H]^+^
7	1.65	Traumatic acid	229.1516	8.2	[M+H]^+^	C_12_H_20_O_4_	Fatty Acyls	58.1 [C_2_H_3_O_2_-H]^+^, 114.1 [C_6_H_9_O_2_]+H^+^, 139.1 [C_10_H_18_]+H^+^, 142.1 [C_8_H_13_O_2_]+H^+^
8	1.84	*p*-Coumaric acid	163.0393	0	[M-H]^−^	C_9_H_8_O_3_	Hydroxycinnamic acids	117.1 [C_8_H_6_O-H]^+^, 119.1 [C_8_H_7_O]^+^
9	1.97	Piperidine	86.06009	0.4	[M+H]^+^	C_5_H_11_N	Piperidines	69.1 [C_5_H_10_-H]^+^
10	2.04	Ferulic acid	193.0497	2.1	[M-H]^−^	C_10_H_10_O_4_	Hydroxycinnamic acids	134.1 [C_8_H_6_O_2_]^−^, 149.1 [C_9_H_9_O_2_]^−^, 178.1 [C_9_H_7_O_4_]-H^−^
11	2.92	Isookanin-7-glucoside	449.0983	21.1	[M-H]^−^	C_21_H_22_O_11_	Flavonoid-7-O-glycosides	259.1 [C_14_H_11_O_5_]^−^, 287.1 [C_15_H_11_O_6_]^−^
12	5.59	Kaempferol-3-O-alpha-L-rhamnoside	431.1888	6	[M-H]^−^	C_21_H_20_O_10_	Flavonoid-3-O-glycosides	179.1 [C_9_H_5_O_4_+2H]^−^, 223.1 [C_10_H_5_O_6_+2H]^−^, 294.01 [C_14_H_16_O_7_-H]-H^−^, 362.1 [C_18_H_17_O_8_+H]^−^, 385.1 [C_20_H_17_O_8_]^−^
13	5.79	Phlorizin	435.1859	3.3	[M-H]^−^	C_21_H_24_O_10_	Flavonoid O-glycosides	258.1 [C_15_H_13_O_4_+H]^−^, 298.1 [C_13_H_14_O_8_]^−^, 389.1 [C_20_H_20_O_8_+H]^−^
14	6.44	Delphinidin-3-O-beta-glucopyranoside	463.0886	−0.1	[M-2H]^−^	C_21_H_21_O_12_	Anthocyanidin-3-O-glycosides	300.1 [C_15_H_10_O_7_]-2H^−^, 354.1 [C_18_H_14_O_8_-2H]-2H^−^, 394.1 [C_18_H_18_O_10_]^−^
15	6.79	Kaempferol-7-neohesperidoside	593.151	0.3	[M-H]^−^	C_27_H_30_O_15_	Flavonoid-7-O-glycosides	285.1 [C_15_H_9_O^6^]^−^
16	6.86	cyanidin-3-O-rutinoside	595.1702	−4.8	[M]^+^	C_27_H_31_O_15_	Anthocyanidin-3-O-glycosides	287.1 [C_15_H_12_O_6_-H]^+^, 449.1 [C_21_H_22_O_11_-H]^+^
17	6.94	Hyperoside (Quercetin 3-galactoside)	465.1897	−3.1	[M+H]^+^	C_21_H_20_O_12_	Flavonoid-3-O-glycosides	85.028 [C_4_H_7_O_2_-H]-H^−^, 303.1 [C_15_H_10_O_7_+H]^−^
18	6.94	Isoquercitrin	465.1053	−2.4	[M+H]^+^	C_21_H_20_O_12_	Flavonoid-3-O-glycosides	145.1 [C6H10O4-H]^+^, 303.1 [C15H9O7+H]+H^+^
19	6.99	Kuromanin (Cyanidin-3-glucoside)	449.1058	5.2	[M]+	C_21_H_21_O_11_	Anthocyanidin-3-O-glycosides	275.1 [C_14_H_9_O_6_+2H]^+^, 287.1 [C_15_H_10_O_6_+H]^+^
20	7.26	Isorhamnetin-3-O-glucoside	477.1035	−0.2	[M-H]^−^	C_22_H_22_O_12_	Flavonoid-3-O-glycosides	314.1 [C_16_H_11_O_7_]-H^−^, 364.1 [C_17_H_15_O_9_+H]^−^, 392.1 [C_18_H_17_O_10_]-H^−^, 432.1 [C_20_H_16_O_11_]^−^
21	7.51	Diosmin	609.1804	1.4	[M+H]^+^	C_28_H_32_O_15_	Flavonoid-7-O-glycosides	301.1 [C_16_H_11_O_6_+H]+H^+^, 463.1 [C_22_H_21_O_11_+H]+H^+^
22	7.53	Rhoifolin	577.1542	2.1	[M-H]^−^	C_27_H_30_O_14_	Flavonoid-7-O-glycosides	269.1 [C_15_H_9_O_5_]^−^, 532.1 [C_25_H_24_O_13_]^−^
23	7.72	Petunidin-3-O-beta-glucopyranoside	479.1093	15.9	[M]^+^	C_22_H_23_O_12_	Anthocyanidin-3-O-glycosides	302.1 [C_15_H_9_O_7_+H]^+^, 317.1 [C_16_H_12_O_7_+H]^+^, 371.1 [C_19_H_16_O_8_-H]^+^
24	7.97	Peonidine-3-O-glucoside	463.1237	1.4	[M]^+^	C_22_H_23_O_11_	Anthocyanidin-3-O-glycosides	301.1 [C_16_H_12_O_6_+H]^+^, 430.1 [C_21_H_19_O_10_-H]^+^, 446.1 [C_22_H_22_O_10_]^+^
25	8.05	Daphnetin	179.1064	0.8	[M+H]^+^	C_9_H_6_O_4_	7,8-dihydroxycoumarins	91.1 [C_6_H_3_O]^+^, 105.1 [C_7_H_4_O]+H^+^, 133.1 [C_8_H_6_O_2_-H]^+^, 161.1 [C_9_H_5_O_3_]^+^
26	11.28	Kaempferide	301.0696	2.9	[M+H]^+^	C_16_H_12_O_6_	Flavonols	258.1 [C_14_H_9_O_5_]+H^+^, 286.1 [C_15_H_9_O_6_]+H^+^

**Table 2:** Effects of pretreatment with methanolic date flesh extract (MDFE) on serum biochemical parameters, and hepatic ADH and NADPH in cisplatin-injected rats.

Serum Biochemical Parameters	Groups						
	Control	DF		Cis		DF/Cis	
		Mean ± SE	Percentage Change	Mean ± SE	Percentage Change	Mean ± SE	Percentage Change
ALT (U/l)	23.09 ± 0.60 ^c^	21.30 ± 0.40 ^c^	−7.80%	31.75 ± 2.30 ^a^	37.50%	27.80 ± 2.00 ^b^	20.30%
AST (U/l)	16.95 ± 2.00 ^c^	13.87 ± 3.00 ^c^	−18.10%	27.57 ± 1.90 ^a^	62.65%	21.79 ± 0.50 ^b^	28.50%
AST/ALT	0.74 ± 0.60 ^a^	0.65 ± 0.40 ^a^	−12.10%	0.86 ± 0.050 ^a^	16.20%	0.79 ± 0.33 ^a^	6.75%
GGT (IU/L)	15.12 ± 1.20 ^c^	14.20 ± 1.20 ^c^	−6.00%	30.90 ± 0.50 ^a^	104.30%	23.75 ± 0.80 ^b^	57.00%
Total protein (mg/dL)	6.69 ± 0.34 ^ab^	7.11 ± 0.08 ^a^	6.20%	5.44 ± 0.41 ^b^	−18.60%	6.23 ± 0.25 ^ab^	−6.87%
Albumin (mg/dL)	4.03 ± 0.17 ^ab^	4.26 ± 0.18^a^	5.70%	2.82 ± 0.13 ^c^	−30.00%	3.44 ± 0.18 ^bc^	−14.60%
Globulin (mg/dL)	2.66 ± 0.10 ^a^	2.62 ± 0.20 ^a^	−1.50%	2.62 ± 0.40 ^a^	−1.50%	2.72 ± 0.30 ^a^	2.25%
ADH (U/g protein)	29.44 ± 0.40 ^a^	29.24 ± 1.40 ^a^	−0.67%	17.27 ± 0.10 ^c^	−41.30%	26.24 ± 1.40 ^b^	−0.10%
NADPH (nmol/mg protein)	10.80 ± 0.30 ^a^	11.54 ± 0.70 ^a^	6.90%	6.00 ± 0.30 ^c^	−44.40%	8.78 ± 0.30 ^b^	−1.73%

Data are presented as means ± SE (*n* = 5). Mean values with different superscript letters within the same row are significantly different at *p* ≤ 0.05 using ANOVA followed by a Tukey multiple comparison test. DF, methanolic date flesh extract; Cis, cisplatin; ALT, alanine aminotransferase; AST, aspartate aminotransferase; ADH, alcohol dehydrogenase; NADPH, nicotinamide adenine dinucleotide phosphate.

**Table 3:** Effect of pretreatment with methanolic date flesh extract (MDFE) on the liver protein carbonyl content (PCC), malondialdehyde (MDA), superoxide dismutase (SOD), reduced glutathione (GSH), interleukin 10 (IL-10), and interleukin 12 (IL-12) in the cisplatin-injected rats.

Liver Biochemical Parameters	Groups						
	Control	DF		Cis		DF/Cis	
		Mean ± SE	Percentage Change	Mean ± SE	Percentage Change	Mean ± SE	Percentage Change
MDA (nmol/g)	0.85 ± 0.16 ^c^	0.73 ± 0.20 ^c^	−14.10%	2.37 ± 0.20 ^a^	178.80%	1.49 ± 0.10 ^b^	75.20%
PCC (nmol/gtissue)	1.73 ± 0.02 ^c^	1.58 ± 0.20 ^c^	−8.60%	4.05 ± 0.10 ^a^	134.10%	2.54 ± 0.30 ^b^	46.80%
SOD (U/g protein)	11.21 ± 0.30 ^b^	12.89 ± 0.50 ^a^	14.90%	7.79 ± 0.02 ^d^	−30.50%	9.82 ± 0.20 ^c^	−12.30%
GSH (mg/g tissue)	18.95 ± 0.11 ^a^	20.46 ± 0.70 ^a^	7.90%	13.50 ± 0.80 ^c^	−28.70%	16.53 ± 0.40 ^b^	−12.70%
IL-10 (pg/mL)	57.75 ± 1.20 ^a^	58.26 ± 0.50 ^a^	0.80%	38.87 ± 1.70 ^c^	−0.56%	43.94 ± 3.10 ^c^	−23.90%
IL-12 (pg/mL)	34.65 ± 1.17 ^c^	32.24 ± 1.81 ^c^	−6.90%	71.97 ± 2.10 ^a^	107.70%	53.10 ± 2.60 ^b^	53.20%

Data are presented as means ± SE (*n* = 5). Mean values with different superscript letters within the same row are significantly different at *p* ≤ 0.05 using ANOVA followed by a Tukey multiple comparison. DF, methanolic date flesh extract; Cis, cisplatin.

**Table 4:** Effect of pretreatment with methanolic date flesh extract (MDFE) on the scoring of liver injury in cisplatin-injected rats.

	Groups				
Histological Changes	Degree	Control	DF	Cis	DF/Cis
Necrosis	1	0.00 ± 0.00	0.00 ± 0.00	3.10 ± 0.30 *	1.10 ± 0.01 *
	2	0.00 ± 0.00	0.00 ± 0.00	6.40 ± 0.50 *	0.00 ± 0.00
	3	0.00 ± 0.00	0.00 ± 0.00	0.00 ± 0.00	0.00 ± 0.00
Hydropic degeneration	1	0.00 ± 0.00	0.00 ± 0.00	2.20 ± 2.20 *	1.00 ± 0.01 *
	2	0.00 ± 0.00	0.00 ± 0.00	11.60 ± 0.20 *	0.00 ± 0.00
	3	0.00 ± 0.00	0.00 ± 0.00	0.00 ± 0.00	0.00 ± 0.00
Fatty degeneration	1	0.00 ± 0.00	0.00 ± 0.00	5.80 ± 1.50 *	0.00 ± 0.00
	2	0.00 ± 0.00	0.00 ± 0.00	1.66 ± 0.70 *	0.00 ± 0.00
	3	0.00 ± 0.00	0.00 ± 0.00	0.00 ± 0.00	0.00 ± 0.00
Infiltration of inflammatory cells	1	0.00 ± 0.00	0.00 ± 0.00	0.00 ± 0.00	1.08 ± 0.01 *
	2	0.00 ± 0.00	0.00 ± 0.00	2.68 ± 0.80 *	0.00 ± 0.00
	3	0.00 ± 0.00	0.00 ± 0.00	8.20 ± 1.11 *	0.00 ± 0.00

Values are presented as means ± SE. (*) denotes a significant variation from the control group (Kruskal–Wallis, *p* ≤ 0.05). Degree: 1, mild; 2, moderate; 3, severe. DF, methanolic date flesh extract; Cis, cisplatin.

## Data Availability

Not applicable.
